# Supplementary material for: Beyond coincidence: An investigation of the interplay between synchronicity awareness and the mindful state
Source: PLoS One. 2024 Oct 14;19(10):e0307443. doi: 10.1371/journal.pone.0307443 (PMC11472937; doi:10.1371/journal.pone.0307443)
Supplement: S1 Appendix — (DOCX) [file pone.0307443.s001.docx]

**Appendix 1**

**Item analyses of the SAMD questionnaire**

In order to further examine the properties of the Synchronicity Awareness and Meaning Detection questionnaire, we submitted SAMD items to the following analyses: Item-Scale properties of the synchronicity awareness items (see Table A) and the meaning detection items (see Table B); interitem correlation for synchronicity awareness items (see Table C), and for meaning detection items (see Table D).

Item-Scale properties:

| Table A.  *Item-scale properties of synchronicity awareness items.* | | | |  |
| --- | --- | --- | --- | --- |
| **Item** | **Cronbach's α if item dropped** | | **Item-rest correlation** |  |
| SA_1 | .775 | | .585 |  |
| SA_2 | .795 | | .433 |  |
| SA_3 | .801 | | .388 |  |
| SA_4 | .786 | | .496 |  |
| SA_5 | .787 | | .491 |  |
| SA_6 | .775 | | .593 |  |
| SA_7 | .776 | | .574 |  |
| SA_8 | .791 | | .458 |  |
| SA_9 | .787 | | .492 |  |
| *Note.* Cronbach’s alpha was .81. | | | |  |
| Table B.  *Item-scale properties of meaning detection items.* | | | | |
| **Item** | | **Cronbach's α if item dropped** | **Item-rest correlation** | |
| MD_1 | | .903 | .640 | |
| MD_2 | | .903 | .646 | |
| MD_3 | | .899 | .723 | |
| MD_4 | | .904 | .602 | |
| MD_5 | | .905 | .594 | |
| MD_6 | | .900 | .712 | |
| MD_7 | | .903 | .646 | |
| MD_8 | | .904 | .616 | |
| MD_9 | | .898 | .742 | |
| MD_10 | | .898 | .735 | |
| MD_11 | | .911 | .421 | |
| MD_12 | | .909 | .474 | |
| MD_13 | | .904 | .617 | |
| Note. Cronbach’s alpha was .91. | | | | |

Interitem correlation matrices:

| Table C.  *Pearson correlation between synchronicity awareness items.* | | | | | | | | |
| --- | --- | --- | --- | --- | --- | --- | --- | --- |
| **Variable** | **SA_1** | **SA_2** | **SA_3** | **SA_4** | **SA_5** | **SA_6** | **SA_7** | **SA_8** |
| SA_1 | — |  |  |  |  |  |  |  |
| SA_2 | .397 | — |  |  |  |  |  |  |
| SA_3 | .249 | .183 | — |  |  |  |  |  |
| SA_4 | .372 | .219 | .271 | — |  |  |  |  |
| SA_5 | .333 | .330 | .221 | .379 | — |  |  |  |
| SA_6 | .469 | .382 | .259 | .383 | .390 | — |  |  |
| SA_7 | .421 | .322 | .274 | .339 | .379 | .382 | — |  |
| SA_8 | .333 | .170 | .306 | .263 | .200 | .361 | .342 | — |
| SA_9 | .361 | .213 | .261 | .288 | .246 | .334 | .428 | .398 |
| *Note*. All correlation coefficients were significant at p<.001. | | | | | | | | |

| Table D.  *Pearson correlation between meaning detection items.* | | | | | | | | | | | | |
| --- | --- | --- | --- | --- | --- | --- | --- | --- | --- | --- | --- | --- |
| **Variable** | **MD_1** | **MD_2** | **MD_3** | **MD_4** | **MD_5** | **MD_6** | **MD_7** | **MD_8** | **MD_9** | **MD_10** | **MD_11** | **MD_12** |
| MD_1 | — |  |  |  |  |  |  |  |  |  |  |  |
| MD_2 | .324 | — |  |  |  |  |  |  |  |  |  |  |
| MD_3 | .538 | .501 | — |  |  |  |  |  |  |  |  |  |
| MD_4 | .471 | .409 | .459 | — |  |  |  |  |  |  |  |  |
| MD_5 | .499 | .350 | .511 | .451 | — |  |  |  |  |  |  |  |
| MD_6 | .471 | .592 | .655 | .452 | .446 | — |  |  |  |  |  |  |
| MD_7 | .402 | .600 | .555 | .416 | .351 | .600 | — |  |  |  |  |  |
| MD_8 | .503 | .378 | .452 | .404 | .437 | .477 | .384 | — |  |  |  |  |
| MD_9 | .520 | .487 | .563 | .497 | .482 | .579 | .492 | .525 | — |  |  |  |
| MD_10 | .512 | .545 | .582 | .450 | .486 | .555 | .537 | .503 | .635 | — |  |  |
| MD_11 | .253 | .394 | .277 | .304 | .282 | .250 | .239 | .298 | .374 | .342 | — |  |
| MD_12 | .377 | .314 | .359 | .318 | .364 | .343 | .289 | .328 | .380 | .419 | .264 | — |
| MD_13 | .484 | .442 | .488 | .411 | .344 | .416 | .448 | .467 | .567 | .477 | .310 | .284 |
| *Note.* All correlation coefficients were significant at p<.001. | | | | | | | | | | | | |

Overall, these analyses reveal good internal consistency in both SAMD factors. However, the EFA highlighted two items, one of synchronicity awareness (i.e., item 3) and one of meaning detection (i.e., item 7), that were not loaded onto their hypothesized factors (or to any other factor). To examine the manner to which the EFA factors differed from the CFA factors, we calculated mean scores to the four factors that did not show EFA-CFA compatibility, namely synchronicity awareness, meaning detection, encoding style and Langer’s mindfulness scale. Pearson correlation matrix is presented in Table E.

| Table E.  *Pearson correlations between EFA factors (columns) and CFA factors (rows).* | | | | | | | | |
| --- | --- | --- | --- | --- | --- | --- | --- | --- |
| **Factors** | **fMD** | | **fSA** | | **fLMS** | | **fESQ** | |
| MD | **.954** | *** | .515 | *** | .355 | *** | .320 | *** |
| SA | .512 | *** | **.985** | *** | .225 | *** | .326 | *** |
| LMStot | .294 | *** | .232 | *** | **.965** | *** | .005 |  |
| ESQ | .336 | *** | .336 | *** | .020 |  | **.989** | *** |
| MAAS | -.094 | * | -.108 | ** | .297 | *** | -.313 | *** |
| FFMQ-Observe | .399 | *** | .282 | *** | .381 | *** | .163 | *** |
| FFMQ-Awareness | -.069 |  | -.066 |  | .228 | *** | -.208 | *** |
| FFMQ-Non-Judging | -.172 | *** | -.065 |  | .130 | ** | -.233 | *** |
| FFMQ-Non-reacting | -.009 |  | .034 |  | .199 | *** | -.114 | ** |
| FFMQ-Describe | .069 |  | .104 | * | .344 | *** | -.142 | *** |
| *Note.*   MD- Meaning Detection; SA- Synchronicity Awareness; FFMQ- Five Facets of Mindfulness Questionnaire; LMStot- Langer’s Mindfulness Scale total score; ESQ- Encoding Style Questionnaire; MAAS-Mindfulness Attention and Awareness Scale; fMD, fSA, fLMS, and fESQ refer to EFA factors of MD, SA, LMStot, and ESQ, respectively; Bolded coefficients are correlations between EFA and CFA corresponding factors; * p < .05, ** p < .01, *** p < .001. | | | | | | | | |

Table E reveals that although some items did not load onto their hypothesized factor, the EFA factors are still very highly correlated with their CFA counterparts.
