# Supplementary material for: Beyond coincidence: An investigation of the interplay between synchronicity awareness and the mindful state
Source: PLoS One. 2024 Oct 14;19(10):e0307443. doi: 10.1371/journal.pone.0307443 (PMC11472937; doi:10.1371/journal.pone.0307443)
Supplement: Attachment — Submitted filename: Response to reviewers PLOS1 Apr2024.docx [file pone.0307443.s002.docx]

**Response to Reviewers**

**We wish to thank both reviewers for their insightful comments. We have made a number of changes to address all the issues raised by the reviewers. The paper is considerably stronger as a result. Below we detail the changes we made in blue.**

**Reviewer #1:** 

The manuscript was submitted to language editing. Corrections were made throughout the manuscript.

1. The study should be framed as one of bringing an aspect of mindfulness, “synchronicity awareness” in relation to other more studied aspects of the greater construct of mindfulness. Otherwise the rationale is not immediately clear from the abstract.

We thank Reviewer 1 for this insightful comment. We have completely rewritten the abstract and we believe it now presents the rationale more clearly.

2. The reason for a dominantly caucasian sample is unclear (given the highly diverse populations available in US and UK), and will limit generalizability. I would strongly recommend recruiting more non-caucasian participants.

We thank Reviewer 1 for this comment. Although the Prolific holds a highly diverse pool of participants, unfortunately most of the people choosing to take part in our study identified as Caucasians. We do agree that our ability to generalize the findings has been limited due to sample characteristics, and we now address this issue in the discussion (see P. 18 top). Nevertheless, following this comment we examined group differences between Caucasians and non-Caucasians, and found that these groups differed solely in Age (Caucasians were 6 years older in average) and Religiosity (Caucasians reported to be less religious). No differences were observed in any of the critical variables of the study.

3. The amount of compensation received by participants is not mentioned. Thank you for your attention. This was added. Please see page 8.

4. Points for improvement in writing (due to the unfortunately high number of incidences of typos, grammatical errors, and awkward or disorganized composition, I could not provide a full list. Please revise considerably before submitting):
Thank you for your attention. Indeed, many typos and grammatical errors were found and hopefully we got them all now.

P.2, L.3 should read” it has scarcely been examined scientifically and little is known…”
This was corrected. Thank you.

P.2, L.6 should read “engaged in an online survey…” Done

P.3, L. 2 should read “mindful state is characterized” Done

P.3. L.5-6 this sentence is awkward , should read something like” mindfulness has been conceived as an acquired skill, and as a trait-like construct” Done

P.3, L.8 should read “multifaceted construct” Done

P.3, L.12, 14, should read “curious” and innovative thought” Done

P.3, L.20, The sentence starting with “for example, mindfulness” needs to be rewritten for consistency of tense (which manifested), and I assume you mean “processing and evoking ‘a feeling of memory’ during…”? Done

P.5, L.18, the sentence beginning with “given that…” is a disorganized, run-on sentence. Please simplify or break up into smaller sentences. Done

P.6 L.19, The last sentence is extremely vague and unnecessary. Potential meeting points and relationships between what? Done

P.9, L.19, should read “Trait socio-cognitive”, i.e. no “A” is necessary. Done

**Reviewer #2:**

1. Given that one of the primary goals was to validate the SAMD questionnaire, it would be beneficial for the authors to conduct additional analyses to assess its reliability. For instance, exploring two subscales correlation, item-subscale correlation, and inter-item correlation could offer more insights.

We now present these analyses in the Appendix. See Tables A and B.

2. They might even consider dividing the results section into two subsections, with one dedicated to SAMD validation and the second to construct validity.

We thank Reviewer 2 for this helpful suggestion. We tried dividing the Results section, but we felt that the outcome was somewhat artificial because the construct validity is an inherent part of the whole validation process. Therefore, we decided to keep the Results in their original format.

3. Additionally, including Cronbach's alpha in the results section rather than the methods section would enhance clarity.

Cronbach’s alphas are now presented in Table 2.

4. It is unclear why the MAAS was not included in the EFA. While I acknowledge the potential redundancy with the FFMQ, I don’t get how it is an issue to test whether SAMD items load on two distinguishable factors, independent of MAAS.

Thank you for this insightful comment. The MAAS was highly correlated with FFMQ’s ‘Acting with awareness’ (Pearson’s r = 0.78). Because EFA is basically a set of multiple regression analyses with are sensitive to multicollinearity, we decided to exclude the MAAS from this analysis. Indeed, scholars have argued that multicollinearity becomes an issue in EFA when it reaches .9 and above, but we decided to approach it with extreme care because the MAAS and FFMQ’s Awareness are not merely highly correlated but are basically the same thing (FFMQ’s Awareness consists of MAAS items).
It should be noted that we examined Reviewer 2’s suggestion, and the analysis revealed better fit indices and is preferable from a purely statistic point of view (e.g., RMSEA = .030 instead of .036; TLI = .904 compared to .875 etc.), but from a methodological point of view it is wrong; Most of the variance of the MAAS has already been accounted for by the FFMQ’s Awareness, resulting in a combined MAAS-FFMQ’s Awareness combination and a few leftovers that do not account for any variance.

5. The decision to provide loading factors for only one of the factors in the EFA raises questions. It would be valuable to observe how different items load on various factors, providing a more comprehensive understanding of the questionnaire's structure.

We apologize, but there seem to be a somewhat misunderstanding. All factor loadings are presented in Table 1. That is, loadings for all 9 factors are presented. We are not sure whether Reviewer 2 received a partial Table 1 or whether we haven't fully understood the reviewer's intention in Comment 5. We will be glad to clarify and to complete any missing information.

6. The sentence, "Nevertheless, one item from each of the following scales, MD, SA, and ESQ, was loaded onto its corresponding factor. For the LMS, three 'engagement' items and one 'novelty producing' item were loaded onto their corresponding factors," lacks clarity regarding which corresponding factor is being referred to. Including all loading factors would enhance the clarity of this statement.

Indeed, there was a typo in this sentence, with the word “not” accidentally omitted, thus entirely changing the meaning of the sentence Please see the corrected sentence on P. 12-13.

7. It would also be interesting to provide the correlations between factors in both the EFA and CFA as they would also inform the relationships between the identified factors.

This is an interesting insight. Thank you. We checked these correlations, and they were extremely high, suggesting both sets of factors to be practically identical. These correlations are now presented in the Appendix. Please see Tables C and D.

8. Typo: Introduction, L2 : "This mindful state is characterized" not "This mindful state characterized" Done
