# Supplementary material for: Beyond coincidence: An investigation of the interplay between synchronicity awareness and the mindful state
Source: PLoS One. 2024 Oct 14;19(10):e0307443. doi: 10.1371/journal.pone.0307443 (PMC11472937; doi:10.1371/journal.pone.0307443)
Supplement: Attachment — Submitted filename: Response to reviewers PLOS1 June2024.docx [file pone.0307443.s003.docx]

**Response to Reviewers**

**We wish to thank Reviewer 2 for taking the time to clarify the comment we misunderstood in the previous revision. Below we detail the changes we made in blue.**

**Reviewer #2:**

1. The authors have addressed all my comments, except for the fifth one regarding loading factors that was not clear. EFA provides a loading factor of each item on each latent variable. The authors only provided loading factors on the designated latent variable (as in CFA). I was wondering why is that ? While I understand the decision to streamline the presentation, providing loading factors for each item on each latent variable could offer a richer understanding of the relationship between SA/MD items and mindfulness/encoding style.

We thank Reviewer 2 for clarifying this issue. We now present EFA factor loadings in Table 1 and CFA factor loadings in Table 2. See pages 12 and 26.
